# Non-AIDS complexity amongst patients living with HIV in Sydney: risk factors and health outcomes

**DOI:** 10.1186/s12981-018-0193-z

**Published:** 2018-03-08

**Authors:** Derek J. Chan, Virginia Furner, Don E. Smith, Mithilesh Dronavalli, Rohan I. Bopage, Jeffrey J. Post, Anjali K. Bhardwaj

**Affiliations:** 1 Albion Centre, South Eastern Sydney Local Health District, Sydney, NSW 2010 Australia; 2 School of Public Health and Community Medicine, Faculty of Medicine, University of New South Wales, Sydney, NSW 2052 Australia; 3 Department of Infectious Diseases, Prince of Wales Hospital, South Eastern Sydney Local Health District, Sydney, NSW 2010 Australia; 4 Prince of Wales Clinical School, UNSW, Sydney, NSW 2051 Australia; 5 University of Sydney, Sydney, Australia

## Abstract

**Objective:**

To assess the prevalence of non-AIDS co-morbidities (NACs) and predictors of adverse health outcomes amongst people living with HIV in order to identify health needs and potential gaps in patient management.

**Design:**

Retrospective, non-consecutive medical record audit of patients attending a publicly funded HIV clinic in metropolitan Sydney analysed for predictors of adverse health outcomes. We developed a scoring system based on the validated Charlson score method for NACs, mental health and social issues and confounders were selected using directed acyclic graph theory under the principles of causal inference.

**Results:**

211 patient files were audited non-consecutively over 6 weeks. 89.5% were male; 41.8% culturally and linguistically diverse and 4.1% were of Aboriginal/Torres Strait Islander origin. Half of patients had no general practitioner and 25% were ineligible for Medicare subsidised care. The most common NACs were: cardiovascular disease (25%), hepatic disease (21%), and endocrinopathies (20%). One-third of patients had clinical anxiety, one-third major depression and almost half of patients had a lifetime history of tobacco smoking. Five predictors of poor health outcomes were identified: (1) *co*-*morbidity score* was associated with hospitalisation (odds ratio, OR 1.58; 95% CI 1.01–2.46; *p* = 0.044); (2) *mental health score* was associated with hospitalisation (OR 1.79; 95% CI 1.22–2.62; *p* = 0.003) and poor adherence to ART (OR 2.34; 95% CI 1.52–3.59; *p *= 0.001); (3) *social issues score* was associated with genotypic resistance (OR 2.61; 95% CI 1.48–4.59; *p *= 0.001), co-morbidity score (OR 1.69; 95% CI 1.24–2.3; *p *= 0.001) and hospitalisation (OR 1.72; 95% CI 1.1–2.7; *p *= 0.018); (4) *body mass index* < 20 was associated with genotypic resistance (OR 6.25; 95% CI 1.49–26.24; *p* = 0.012); and (5) *Medicare eligibility* was associated with co-morbidity score (OR 2.21; 95% CI 1.24–3.95; *p* = 0.007).

**Conclusion:**

Most HIV patients are healthy due to effective antiretroviral therapy; however, NACs and social/mental health issues are adding to patient complexity. The current findings underpin the need for multidisciplinary management beyond routine viral load and CD4 count monitoring.

## Background

The quality of life and life expectancy of people living with HIV infection (PLHIV) are now similar to that of the general population due to effective antiretroviral therapy (ART) [1]. Whilst ART-related co-morbidities such as lipodystrophy syndrome associated with first-generation nucleosides and protease inhibitors do not occur with the newer agents such as integrase inhibitors, non-AIDS co-morbidities (NACs) such as cardiovascular disease, liver disease and malignancy are increasing among PLHIV [1]. Possible reasons include ageing, ongoing low-level immune activation and inflammation despite virological suppression [2] higher rates of modifiable risk factors (such as smoking) and effects of ART itself [3]. Consequently, HIV management now requires holistic management of NACs, poly-pharmacy and identification of drug–drug interactions, as well as attention to psychosocial issues that may affect ART compliance.

Australian PLHIV are managed by accredited general practitioners (S100 GPs) and hospital/clinic doctors; however, in our experience marginalised, complex patients (e.g. culturally and linguistically diverse (CALD), aboriginal/torres strait islander (ATSI) patients, those with complex mental health, substance use, socioeconomic problems, or those without federally-funded healthcare (Medicare) may not see GPs due to cost, confidentiality concerns, and lack of appointments or multidisciplinary/multicultural support. The public health system provides care for individuals who, if unlinked to private health care, may potentially become immune-compromised, require hospitalisation, or transmit HIV. Data on the health complexity of this important group is scarce. We assess the prevalence of NACs and predictors of adverse health outcomes for HIV patients attending a publicly funded ambulatory care clinic.

## Methods

### Setting and participants

The Albion Centre is a publicly funded, ambulatory care clinic in metropolitan Sydney with in-house medical specialists, nurses, psychologists, social workers, dieticians and pharmacists providing same-visit HIV care and treatment. The patients are mostly homosexually-active men, the group most affected by Australia’s HIV epidemic.

### Design

A checklist was developed to enable a retrospective, non-consecutive audit of medical records for HIV patients presenting for medical care between 1 May–15 June 2014. The project was approved by South Eastern Sydney Human Research Ethics Committee (study number 16/215) adjust.

Data collected included; (1) patients’ demographic information, including gender, age, CALD or ATSI background, GP or specialist involvement in care; (2) clinical history; duration of HIV infection, recent HIV RNA viral load, current and nadir CD4 count, HIV genotypic resistance, ART adherence, past AIDS defining conditions, hospital admissions in past year, NACs (cardiac, hepatic, endocrine, renal, neurological, respiratory, neoplastic, musculoskeletal conditions), body mass index (BMI); and (3) psychosocial factors; such as mental health diagnoses, smoking, alcohol and (intravenous) drug use, employment status, housing, financial support and federally-funded Medicare eligibility (only Australian permanent residents and citizens are eligible for Medicare). Regarding categorisation where a particular condition could be reported under more than one NAC domain (e.g. haemochromatosis could be classified as liver, endocrine or cardiac disease) only one NAC was assigned. We did not adjust for frequency of attendance as this was a cross-sectional, non-consecutive analysis of patient records. The mental health diagnoses were made by the attending doctor with or without input from staff clinical psychologists. ATSI and CALD status were defined according to patient registration data including self-declaration of aboriginality, first language spoken at home and year of arrival in Australia if born overseas. The designation of ‘social isolation’ was derived from the patient’s psychosocial history and self-report of same as documented in the records. Refugees were patients who were referred by the Australian Immigration authorities for treatment and care.

### Data analysis

**Fig. 1:**
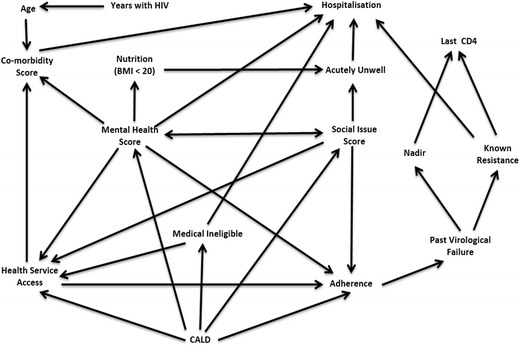
Pre-analysis direct acyclic graph for HIV patient complexity

Data were analysed using Stata software (StataCorp. 2011, v12: College Station, TX: StataCorp LP). DAG theory was used to determine confounders for each predictor-outcome association (Fig. 1). DAG theory minimises bias and identifies only necessary confounders to increase the power of the analysis (statistical efficiency) [4]. The adverse health outcomes identified in our study were: CD4 count, genotypic resistance, hospital admission, ART adherence and co-morbidity score (health scores are discussed below).

**Fig. 2:**
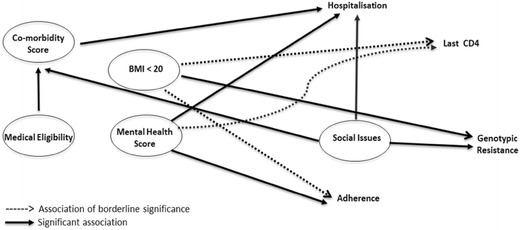
Post-analysis direct acyclic graph: predictors of adverse health outcomes for HIV patients

Figure 1 shows the pre-analysis DAG for HIV patients. Arrows in the DAGs in this paper (Figs. 1 and 2) represent a causal association as per syntax of causal inference used by Pearl [5]. This model is not strictly acyclic, as mental health diagnoses may cause social issues and vice versa. For these ‘bi-directional’ variables, the social issue score was considered a confounder for the mental health score and vice versa in the analysis. This produces conservative effect sizes when assessing causality but the probability rules of the model are maintained.

Associations between predictors with binary outcomes were calculated using logistic regression. The exception is ordinal regression, which is used to calculate the associations between the co-morbidity score and CD4 nadir, social issue score, Medicare eligibility, virological failure, BMI > 30 and CALD. Missing values were ignored (case-wise deletion) and multiple imputation was not performed because some variables in the dataset had more missing observations than others and were likely missing not at random (MNAR) making them unsuitable for multiple imputation.

We developed ‘health scores’ to facilitate quantitative analysis. These scores, although not validated for PLHIV, follow the principles of the validated Charlson score [6] and are simply a method for counting the total number or NACs, mental health diagnoses and social issues for each patient. The Comorbidity score was the number of NACs (zero to a maximum seven defined co-morbidities including Cardiac, Renal, Neurological, Liver, Endocrine, Respiratory, Metabolic Bone Disease, Malignancy). The mental health score was the sum of mental health diagnoses (zero to a maximum three diagnoses including anxiety, major depression, drug-dependency, alcohol dependency, personality disorder, schizophrenia and other psychoses). Lastly, the social issues score was the sum of social issues (zero to a maximum of three issues including: unemployment, disability support pension, socially isolated, refugee, retired, homeless, domestic violence).

## Results

### Demographics

211 files of 264 attendees had audit forms completed in the study period. 89.5% were male, 10% female and 0.5% transgender. Median age was 47.2 years (range 21–73). Many patients were CALD (41.8%) and 4.1% ATSI 95% were taking ART and one-third had HIV infection for 20 years or more. All patients are requested to have a GP as part of our shared-care model, yet only half did. The reasons patients reported for not having a GP included: fear of disclosure, limited GP appointments, cost or lack of multidisciplinary services, or perceived lack of HIV expertise. Half had a specialist managing their NACs. Variables from the DAG model and health scores are given in Table 1.

**Table 1:** Variables from direct acyclic graph and health scores

	1	0	Yes	Proportion	
				Yes	Total
Predictor/confounder					
CD4 nadir < 200 cells/µL	Yes	No	67	0.4	169
Medicare eligible	Yes	No	157	0.75	210
Virological failure	Yes	No	27	0.14	193
BMI < 20	Yes	No	26	0.23	112
BMI > 30	Yes	No	28	0.25	112
CALD	Yes	No	87	0.42	208
Outcomes					
Last CD4 (cells/µL)	< 200	> 200	74	0.36	206
Genotypic resistance	Yes	No	18	0.1	186
Hospitalisation past 12 months	Yes	No	36	0.17	209
Adherence issue	Yes	No	38	0.18	211

Comorbidity score:	From 0 to 7				
	0		90	0.43	211
	1		60	0.28	211
	2		34	0.16	211
	3		16	0.08	211
	4		5	0.02	211
	5		3	0.01	211
	6		2	0.01	211
	7		1	0.005	211

Mental health score:	From 0 to 3				211
	0		97	0.47	
	1		66	0.32	
	2		34	0.16	
	3		13	0.06	

Social issue score:	From 0 to 3				211
	0		57	0.27	
	1		103	0.49	
	2		35	0.17	
	3		16	0.08	

### Clinical parameters

The patients were generally healthy: 94% were taking ART and 82.3% had undetectable viral load. 18% had poor ART adherence (as determined by doctor), 8.1% past genotypic resistance and 12.8% past virological failure. 12.4% were underweight (BMI < 20) and 12.9% were obese (BMI > 30). 17% had been hospitalised in the past 12 months (most not HIV-related) and 13% had a past AIDS-defining condition. Polypharmacy was common with 20% prescribed five or more non-ART medications. Approximately one-third had been living with HIV for 10–20 years and one-third more than 20 years.

### Non-AIDS comorbidities

43% of patients had no NAC. 28.1% had at least one NAC and some had multiple NACs. The prevalence of specific NACs (with predominant condition) was: cardiovascular disease (hypercholesterolaemia, hypertension) 26.8%; liver disease (hepatitis B or C co-infection, alcoholic liver disease) 22.1%; endocrinopathy (e.g.: type 2 diabetes, dyslipidemia) 21.7%; neurological disease (peripheral neuropathy, HIV-associated neurocognitive disorder) 12%; renal impairment (eGFR < 60 mL/min) 7.8%; osteoporosis 6%; respiratory disease (chronic obstructive pulmonary disease) 6.9%; and malignancy 4.6%.

### Psychosocial issues

There was a significant burden of mental health and social issues in this sample. 54% had at least one psychiatric diagnosis: predominantly anxiety disorder (one-third) and major depression (one-third), followed by drug dependence (12.9%), alcohol dependence (4.3%), personality disorder (3.7%), schizophrenia (3.1%), other psychosis, e.g. drug-induced (3.1%), post-traumatic stress disorder (2.4%), bipolar affective disorder (1.8%) and adjustment disorder (1.8%). Dual mental health diagnoses occurred in 20% of patients. 49% were past/current smokers, 14.3% were past or current intravenous drug users, and 1.8% were homeless. About one-fifth of patients were unemployed (22.1%), receiving disability support pension (22.6%) or were Medicare ineligible (25%).

### Predictors of poor health outcomes

We identified five predictors of poor health outcomes for our HIV patients adjusting for appropriate confounders as per DAG (Table 2).

**Table 2:** Logistic regression for associations between predictors and outcomes

	OR (95% CI)	*p* value	L 95% CI	U 95% CI	Confounders
Last CD4					
CD4 nadir	3.13 (1.62–6.05)	0.001	1.62	6.05	Crude
ART adherence	3.3	0.001	1.59	6.87	Crude
Mental health score	1.35	0.081	0.96	1.88	Social issue score, CALD
BMI < 20	2.61	0.062	0.95	7.15	ARV adherence
Genotypic resistance					
Virological failure	421.33	0.001	47.7	3721.87	Crude
Social issue score	2.61	0.001	1.48	4.59	CALD
BMI < 20	6.25	0.012	1.49	26.24	Crude
ART adherence	6.64	0.001	2.38	18.53	Crude
Hospital admission					
Co-morbidity score	1.58	0.044	1.01	2.46	Medicare eligibility, mental health score, CALD, social issue score, ART adherence, BMI > 30
Social issue score	1.72	0.018	1.1	2.7	ART adherence, CALD, mental health score
Age	1.04	0.03	1	1.07	Crude
CALD	0.31	0.008	0.13	0.74	Crude
Mental health score	1.79	0.003	1.22	2.62	CALD
Poor ART adherence					
CD4 nadir	2.15	0.064	0.96	4.82	Crude
Virological failure	12.75	0.001	5.1	31.9	Crude
BMI < 20	2.65	0.077	0.9	7.81	Social
Mental health score	2.34	0.001	1.52	3.59	CALD, social issue score
Comorbidity score^a^					
CD4 nadir	2.28	0.005	1.28	4.05	ART adherence
Social issue score	1.69	0.001	1.24	2.3	CALD, mental health score
Medicare eligibility	2.21	0.007	1.24	3.95	CALD
Virological failure	3.15	0.006	1.39	7.13	ART adherence
BMI > 30	3.59	0.002	1.59	8.11	Social issue score, mental health score
CALD	0.62	0.064	0.37	1.03	Crude

*OR* odds ratio; *L95% CI* lower limit, 95% confidence interval; *U95% CI* upper limit, 95% confidence interval; *BMI* body mass index; Crude adjustment for confounding not required; *CALD* culturally and linguistically diverse background

^a^Calculated by ordinal regression

Firstly, the comorbidity score was causally associated with hospitalisation in the previous year, odds ratio, OR 1.58; 95% CI 1.01–2.46; *p* = 0.044 (adjusting for Medicare eligibility, mental health score, social issue score, CALD, ART adherence, BMI > 30 as confounders). Interestingly, CALD status was causally associated with a lower risk of hospitalisation, OR 0.31; 95% CI 0.13–0.74; *p* = 0.008.

The mental health score was also causally associated with hospitalisation, OR 1.79; 95% CI 1.22–2.62; *p* = 0.003 (adjusting for CALD) and poor ART adherence, OR 2.34; 95% CI 1.52–3.59; *p *= 0.001 (adjusting for social issue score, CALD).

The social issue score was causally associated with: (1) hospitalisation, OR 1.72; 95% CI 1.1–2.7; *p *= 0.018 (adjusting for ART adherence, CALD, mental health score); (2) genotypic resistance, OR 2.61; 95% CI 1.48–4.59; *p *= 0.001 (adjusting for CALD); and (3) the co-morbidity score, OR 1.69; 95% CI 1.24–2.3; *p *= 0.001 (adjusting for CALD, mental health score).

A low BMI was causally associated with genotypic resistance, OR 6.25; 95% CI 1.49–26.24; *p* = 0.012 (adjusting for CALD).

Finally, Medicare eligibility was causally associated with the co-morbidity score, even when adjusting for CALD as a confounder, OR 2.21; 95% CI 1.24–3.95; *p* = 0.007.

The final DAG model shows the significant predictors of adverse health outcomes supported by our data (Fig. 2). Notably, it identifies potential specific roles for multidisciplinary management of health predictors in virologically stable patients, underpinning our current model of HIV care.

## Discussion

An audit of PLHIV attending our service revealed a significant number of NACs within this population. A mixture of medical conditions, mental health and social issues reflect the complexity of current management for this population. Within the Australian HIV Observational Database cohort, a recent review of people seen predominantly in general practice showed similar levels of NACs, with rates of depression and anxiety of 35 and 18%, respectively—which is consistent with our findings [7]. As expected in a publicly funded service such as ours, there is an over-representation of Medicare ineligible PLHIV and those with social disadvantage.

Similar scoring methods to assess patient complexity are used elsewhere in medicine. The Comprehensive Geriatric Assessment, for example, is ‘a multidimensional, interdisciplinary diagnostic process to determine the medical, psychological, and functional capabilities of a frail elderly person in order to develop a coordinated and integrated plan for treatment and long-term follow-up’ [8] and a global frailty scoring system for use in the elderly is well recognised [9]. In relation to PLHIV, our development of co-morbidity, mental health and social scores to quantify HIV patient complexity is similar to work conducted by Guaraldi et al. [10] who have developed scoring indices that specifically assess frailty amongst PLHIV.

DAGs can provide an understanding of the complex interrelationships between clinical, psychological and social factors with specific health outcomes and their use is well recognised in modern epidemiology [4]. DAGs have been used widely throughout clinical medicine to identify the minimum set of confounders in order to reduce bias in studies of assessing causation. Examples include nephrology [11], cardiology [12], feto-maternal medicine [13] and orthopaedics [14]. To our knowledge we are the first group to use DAG theory in a study of PLHIV. Our DAG analysis selected confounders and identified five predictors of poor health outcomes within our cohort: i.e., higher comorbidity score, higher mental health score, low social issue score, low BMI and eligibility for Medicare. The association between having a comorbidity and poor health outcomes seems logical, as does mental health issues and poor health outcomes where ARV compliance and health seeking behaviour may be compromised. The association between social deprivation and poor health is also well known across a range of chronic diseases. However, low BMI is difficult to explain when increasing BMI within the general population is associated with a number of chronic diseases such as diabetes, hypertension and cardiovascular disease. This finding may be related to past HIV wasting syndrome or current food insecurity and malnourishment amongst our patients.

The protective effect of being ineligible for Medicare and of CALD status against hospitalisation may seem paradoxical. It is likely that cost of diagnostic tests and hospitalisation are a barrier for our non-Medicare, CALD patients to attending hospital for all except life threatening events, or a reflection of these patients being generally younger within our cohort.

Limitations of this study include that it was conducted at a single site, possible sampling bias due to more unwell patients with NACs perhaps presenting more frequently to our service and not a GP and relatively small sample size and lack of an intervention to test the hypotheses generated. However, by using DAGs only the minimum set of confounders required were used in the analysis making out analyses sample size efficient. The method by which patient records were audited cannot eliminate selection bias but we believe our non-consecutive approach makes this less plausible. Also the complexity score has not been previously validated for use in HIV patients but follows the general principles of the Charlson score in that it accumulates points by body system. The complexity score also summates points without weightings for particular body systems in order to be transparent, easily interpretable and not data driven [6]. Our results cannot, however, be generalised to the entire Australian HIV cohort due to the limitations of the study and the particular patient demographics seen in our clinic.

The study demonstrated associations between mental health and social issues with medical outcomes, suggesting an ongoing need for a biopsychosocial model of multidisciplinary care to overcome the potential disadvantage of PLHIV.

## Conclusion

Although ART has been simplified so that most patients need only take a single, effective and well-tolerated tablet per day, contemporary HIV management by clinicians is not simply a matter of prescribing ARVs and obtaining periodic blood tests for essentially healthy patients. PLHIV are living much longer and, on a background on chronic immune activation, are at increased risk for developing NACs from the interplay of medical, psychological and social factors that may influence long term health [15]. Mental health and social issues may adversely affect adherence to ARVs and clinic attendance, and lifestyle factors such as smoking, alcohol and recreational drug use can further affect the development and progression of some NACs such as cardiovascular disease. Publicly-funded HIV services play a pivotal role in providing a ‘safety net’ for disadvantaged and vulnerable PLHIV who cannot access private health care and require ARV and management of NACs. Engaging individuals with HIV in complex care management may reduce hospitalisation from HIV-related complications and prevent ongoing transmission.
